# Unravelling the Biology of Adult Cardiac Stem Cell-Derived Exosomes to Foster Endogenous Cardiac Regeneration and Repair

**DOI:** 10.3390/ijms21103725

**Published:** 2020-05-25

**Authors:** Teresa Mancuso, Antonella Barone, Alessandro Salatino, Claudia Molinaro, Fabiola Marino, Mariangela Scalise, Michele Torella, Antonella De Angelis, Konrad Urbanek, Daniele Torella, Eleonora Cianflone

**Affiliations:** 1 Molecular and Cellular Cardiology, Department of Experimental and Clinical Medicine, Magna Graecia University, 88100 Catanzaro, Italy; tmancuso@unicz.it (T.M.); barone@unicz.it (A.B.); salatino@unicz.it (A.S.); claudiamolinaro.94@gmail.com (C.M.); marino@unicz.it (F.M.); m.scalise@unicz.it (M.S.); urbanek@unicz.it (K.U.); 2 Department of Translational Medical Sciences, AORN dei Colli/Monaldi Hospital, University of Campania “L. Vanvitelli”, Via Leonardo Bianchi, 80131 Naples, Italy; michele.torella@unicampania.it; 3 Department of Experimental Medicine, Section of Pharmacology, University of Campania “L.Vanvitelli”, 80121 Naples, Italy; antonella.deangelis@unicampania.it; 4 Molecular and Cellular Cardiology, Department of Medical and Surgical Sciences, Magna Graecia University, 88100 Catanzaro, Italy; cianflone@unicz.it

**Keywords:** heart, exosomes, extracellular vesicles, cardiac progenitor cells, cardiac stem cells, cardiac regeneration

## Abstract

Cardiac remuscularization has been the stated goal of the field of regenerative cardiology since its inception. Along with the refreshment of lost and dysfunctional cardiac muscle cells, the field of cell therapy has expanded in scope encompassing also the potential of the injected cells as cardioprotective and cardio-reparative agents for cardiovascular diseases. The latter has been the result of the findings that cell therapies so far tested in clinical trials exert their beneficial effects through paracrine mechanisms acting on the endogenous myocardial reparative/regenerative potential. The endogenous regenerative potential of the adult heart is still highly debated. While it has been widely accepted that adult cardiomyocytes (CMs) are renewed throughout life either in response to wear and tear and after injury, the rate and origin of this phenomenon are yet to be clarified. The adult heart harbors resident cardiac/stem progenitor cells (CSCs/CPCs), whose discovery and characterization were initially sufficient to explain CM renewal in response to physiological and pathological stresses, when also considering that adult CMs are terminally differentiated cells. The role of CSCs in CM formation in the adult heart has been however questioned by some recent genetic fate map studies, which have been proved to have serious limitations. Nevertheless, uncontested evidence shows that clonal CSCs are effective transplantable regenerative agents either for their direct myogenic differentiation and for their paracrine effects in the allogeneic setting. In particular, the paracrine potential of CSCs has been the focus of the recent investigation, whereby CSC-derived exosomes appear to harbor relevant regenerative and reparative signals underlying the beneficial effects of CSC transplantation. This review focuses on recent advances in our knowledge about the biological role of exosomes in heart tissue homeostasis and repair with the idea to use them as tools for new therapeutic biotechnologies for “cell-less” effective cardiac regeneration approaches.

## 1. Introduction

Among cardiovascular disease (CVD), myocardial infarction (MI) and heart failure (HF) represent the most important heart conditions in terms of mortality, morbidity and average life expectancy [1,2,3]. Their most significant issue is attributable not to acute mortality but nonfatal cardiovascular events and their long-term consequences. To date, the prevention and the treatment of chronic heart failure (CHF) are based on pharmacotherapy and implantation of medical devices and/or surgical interventions, to maintain or improve the performance of the pre-existing cardiomyocytes (CMs), but not to replace the injured heart with a new and functional cardiac muscle cell population, although CHF is always due to a deficit of cardiomyocytes mass and function. For this reason, for more than three decades by now, the search has been on for an effective therapy capable of refreshing and replacing the significant cardiomyocyte loss and dysfunction after MI and HF [4,5].

For a long time, the heart has been viewed as a postmitotic organ; because of its parenchymal contractile cells, the CMs, in the early postnatal period lose the ability to divide, irreversibly leaving the cell cycle and becoming terminally differentiated cells [6,7,8]. On the contrary, it has been eventually demonstrated that there is a measurable adult CM turnover throughout adult life, although the rate and the significance of this turnover remain controversial [9,10]. Several studies in the past two decades have proposed three main sources of new CMs: (1) circulating progenitors from bone marrow, which through the bloodstream reach the myocardium and there differentiate into cardiomyocytes; (2) pre-existing CMs that can divide and multiply themselves through mitosis and (3) resident/endogenous myocardial multipotent stem cells, able to proliferate and differentiate into the three main cell types of the heart: CMs, vascular endothelial and smooth muscle cells [11,12,13]. Which of the three sources is more relevant or whether indeed they all represent stages of the same phenomena is yet to be finally ascertained.

In the last two decades, the potential use of pluripotent and adult stem cells, and their derivatives for the treatment of various acute and chronic and complex diseases, has witnessed a growing interest in the international scientific enterprise. Several studies suggested that stem/progenitor cell transplantation stimulates tissue repair and regeneration in various organs, including the heart [11,13,14,15,16,17,18,19,20,21]. Since the first discovery of endogenous adult cardiac stem/progenitor cells (CSCs/CPCs), the contribution of these cells to the adult heart has been intensively investigated and nowadays their potential regenerative/reparative role has gained significant interest in the cardiovascular scientific community despite their exact role in endogenous CM renewal during adult life remains to be fully elucidated [13,18,22,23,24,25,26,27,28,29,30].

A plethora of different cell formulations (some of them including different forms of progenitor cells) are being evaluated in clinical trials with some modest and still debated results [31,32,33,34]. Indeed, although some beneficial effects have been observed in patients with chronic heart failure and refractory angina, cell transplantation, as applied so far, is not sufficient to promote effective cardiac regeneration [35,36]. Among other issues, the low survival rate of transplanted cells limits the therapeutic efficacy of cell therapy [37,38]. Not less significant remains the issue of the cost of autologous stem cell therapy that carries the additional and significant limitation of not being available at the time of MI when indeed cell therapy would be most needed to reduce the burden of cell damage and death.

To circumvent the above problems, which are inherent to autologous stem cell therapy, it has been proposed the use of allogeneic stem cells as a potential therapeutic approach to foster direct remuscularization of the damaged heart or its indirect repair through the stimulation of the endogenous regenerative process [39,40,41,42]. The first option has been successfully tested by the transplantation of hESC-derived cardiomyocytes in non-human primates using low dose immunosuppression [39,40,41]. However, despite the positive results of such an approach, it remains questionable to envision a future of millions of patients with MI and post-MI HF treated with allogenic cells undergoing lifelong immunosuppression with all the potential side effects of a similar clinical scenario. On the other hand, the idea of allogeneic stem cells without immunosuppression has been promoted by the evidence that actually autologous transplanted cells do not engraft, are short-lived and manly act through paracrine mechanisms to promote repair and regeneration of the damaged tissue at target [43,44,45]. Indeed, both allogeneic mesenchymal stem cells and cardiac stem cells, alone or in combination have been tested in preclinical models with positive results and also reached the first-in-man clinical experimentation [43,44,45]. The latter has created the path for the idea of creating “supercargo cells”, which is transplantable cellular reservoirs engineered to release multiple cardioprotective and cardio-regenerative factors or small molecules to obtain effective myocardial regeneration [45,46].

Notwithstanding the above approaches that rely on the use of cells, recent evidence has shown that, upon cell transplantation, cellular crosstalk between transplanted and endogenous cells is a key step for the reparative process [47]. Indeed, it has been proposed that several factors, released from transplanted cells within the host injured tissue, regulate multiple cell processes [48,49]. Among these factors, extracellular vesicles (EVs), and in particular the exosomes released from different types of stem cells, have recently gained burgeoning interest. The latter has generated the field of cell-free myocardial regeneration whereby it is envisioned that using these cell products might be sufficient to foster proper myocardial regeneration for CVDs [48,49]. Cell-free approaches may be advantageous because they bypass many issues related to (stem) cell therapy. The conceptual advantage is that they may be produced in industrial quantities and stored at the point-of-care for off-the-shelf application, ideally without eliciting a relevant recipient immune response or other adverse effects associated with viable cells. These new approaches also do not have risk factors associated with (stem) cell use, like the origin of the cells, the differentiation and proliferation capacity as well as their transformation risk, or their storage, handling and transport conditions.

Recently, there has been a burgeoning interest in EVs and exosomes produced and released by CSCs [50,51]. CSC-derived exosomes can be indeed considered as pivotal elements that can support cardiac tissue homeostasis improving the endogenous repair/regenerative process within the myocardium, displaying also cardioprotective function [50,51,52,53]. Therefore, a better understanding of the nature and mechanisms of action of the CSC-derived exosomes and their function as potential intercellular communicators between CSCs themselves and the surrounding myocardial environment could potentially foster the development of new therapeutic strategies to fight CVDs.

With this premise, in this review, we summarize recent evidence on the role of EVs in cell-to-cell communications within the myocardium and in the cellular response to damage, together with their ability to activate a regenerative response in resident CSCs. Particularly, we focus on recent advances in our knowledge about the biological role of CSC-derived exosomes in heart tissue homeostasis and repair with the idea to use them as tools for new therapeutic biotechnologies for “cell-less” effective cardiac regeneration approaches.

## 2. Resident Cardiac Stem/Progenitor Cells in Adult Myocardial Tissue Homeostasis and Response to Injury

Since 2003, it has been reproducibly shown that the adult heart harbors a population of CSCs that participate in cardiac responses to injury and physiological CM turnover during the lifespan [11,13,14,19,21,54,55,56,57,58]. CSCs cells are clonogenic, self-renewing and multipotent, giving rise to a minimum of three different cardiogenic cell lineages (myocytes, smooth muscle and endothelial cells) both in vitro and in vivo and exhibit significant cardiac tissue regenerative capacity [18,19,20,21].

Unfortunately, to date, there is still a significant controversy in the scientific enterprise over the endogenous role and the endogenous myogenic potential of CSCs in myocardial homeostasis and after injury [24,25,26,59]. The identification and characterization of endogenous CSCs in the adult mammalian heart in 2003 [11] have been initially associated with the expression of c-kit (a type III receptor tyrosine kinase—also named as CD117 or SCF-R, stem cell factor receptor). However, in the adult myocardium, the detection of c-kit *per se* is inadequate and confusing to identify a specific CSC population among all the other c-kit positive (c-kit^pos^) cardiac cells. Indeed, for the vast majority (~90%) of labelled cells, c-kit, as a cell marker within the adult heart tissue, identifies endothelial and mast cells. On the other hand, only less than 10% of the c-kit positive cardiac cells contain multipotent cells [19,20,58,60]. The latter can be enriched by CD45 and CD31 negative sorting from the total c-kit positive cardiac cells. This CD45/CD31^neg^c-kit^pos^ cardiac cell pool is enriched for CSC potential but this three marker-based prospective identification still identifies a heterogeneous cell population, where only 10–20% of these cells are clonogenic/multipotent in vitro and in vivo [19,20,58,60]. Overall, only ~2% of the entire c-kit positive cells fulfil the criteria for multipotent CSCs. When taken at a face value, this evidence suggests that c-kit is indeed a poor biomarker for detecting CSCs within the adult myocardium. However, it is also fundamental to point that c-kit negative cardiac cells do not harbour clonogenic/multipotent cells and *c-kit* haploinsufficiency reduces cardiomyocyte refreshment in the adult heart [11,19,21], which shows that c-kit is not sufficient but yet necessary to identify CSCs [19,20]. The significant heterogeneity within c-kit-labelled cardiac cells has prompted and spread the confusion over the identity and regenerative role of endogenous CSCs. Targeting c-kit as a single marker, murine genetic fate map strategies based on the Cre/Lox recombination have shown to be able to label more than 80% of c-kit-expressing cells in different known *c-kit* domains in the adult mouse [21,29,61]. On that premise, using these tools, the authors of the studies employing this technology assessed the adult hearts claiming that only a minimal number of cardiomyocytes are derived from c-kit-expressing progenitors in adult life [24,25,59,62]. However, we show that this technology fails to fate map CSCs in the adult heart because only less than 10% of the CSC-enriched CD45/CD31^neg^c-kit^pos^ are labelled in these c-kit^Cre^ mice [29]. Furthermore, CRE knock-in causes *c-kit* haploinsufficiency producing a significant deficit in the myogenic potential of CSCs in vitro and in vivo [21,29,61]. Therefore, appropriate genetic fate map strategies, able to actually label CSCs in vivo, are still needed to address the myogenic role of CSCs in vivo.

The controversy and debate over the myogenic role of resident CSCs have been awkwardly fueled by the recent retractions of several papers by one of the scientists mainly involved with the discovery and characterization of this cell entity [63]. It is a fact that the scandal surrounding those retracted publications has scored a significant setback for the field of resident CSC biology and regenerative potential [63,64]. However, it must be remembered that it would be equally twisting for this field if because of those misdeeds all the independent and reproducible investigations on the regenerative role of CSCs were dismissed [63,64]. It is worth here remembering that independent groups have contributed to the identification and characterization of adult resident CSCs [14,65,66,67,68], whose publications have never been questioned or retracted.

Aside from the above scandal, which is not the topic of this review, the identity of true CSCs has been revealed by the analysis of the clonal population derived from CD45/CD31^neg^c-kit^pos^ cardiac cells. RNASeq and FACS analysis of these CSC clones show that CSCs express different and well-characterized membrane markers such as Sca-1, Abcg-2, Flk-1, CD105, CD166 and PDGFR-α and several cardiac embryonic transcription factors (Tert, Bmi-1, Gata-4, Mef2c and Nkx2.5) [19,60,69,70,71]. These clonal cells are robustly myogenic in vitro and in vivo. Therefore, these data incontrovertibly show that CSCs are potent myogenic precursors with a significant cardiac remuscularization potential when transplanted in vivo.

In the adult heart, endogenous CSCs are nested within both atrial and ventricular myocardium, with the highest density in the apex and the atria [54,72]. In typical adult stem cell systems, the niche influences adult stem cell behavior, through their interaction with the parenchymal stromal cell types and the interaction between the stem cells themselves [73]. The complex network of signals within this dynamic structure appears to be responsible for the maintenance of a balance from stemness to differentiation and therefore for the integrity of the adult stem cell pool within the relative tissue. The latter is set to allow for an adequate tissue response and homeostasis in physiological and pathological conditions, with a leading role in the repair process after damage [72,73].

These issues have driven the interest of the scientific community toward banked, consistent and readily available allogeneic cell products, in which cells derived from healthy donors or unaffected organs can be strictly quality-controlled and manufactured in large quantities in a central facility to be available at any time as an “off-the-shelf” product, also useful for clinical emergencies including MI. The main advantage of this approach is that the functional variability of the cells is alleviated by preparing a master bank of validated fully-tested clinical-grade clonal cells that allows a well-qualified product to be readily available for a range of clinical applications On the other hand, as tissue-specific stem/progenitor cells, endogenous CSCs, when *ex-vivo* expanded and reinjected through the coronary circulation, show a high tropism and engraftment within injured myocardium, which appears higher than any other cell injected [74,75]. All the above prompted the test of allogeneic CSCs in the setting of acute myocardial infarction in animal models. Allogeneic CSCs indeed secrete a plethora of cardiopoietic factors as well as exerting a significant role in modulating the myocardial immune response within myocardial injured tissue [39,76]. Allogeneic, nonmatched, cloned male EGFP-transduced porcine eCSCs, were administered intracoronary in white Yorkshire female pigs, 30 min after MI and coronary reperfusion [77]. Pig serum was injected to control pigs after MI (CTRL). The cells or sera were injected through a percutaneous catheter into the anterior descending coronary artery just below the site of balloon occlusion used to produce the AMI. We found a high degree of EGFP^pos^/c-kit^pos^ heterologous HLA nonmatched allogeneic porcine CSCs nesting in the damaged pig myocardium at 30 min through to one day after MI. At three weeks post-AMI, all the injected allogeneic cells had disappeared from the myocardium and peripheral tissues (i.e., spleen). There was significant activation of the endogenous GFP^neg^ c-kit^pos^ CSCs (eCSCs) following allogeneic CSC treatment, so that by three weeks after MI, there was increased new cardiomyocyte and capillary formation, which was not evident in the control hearts. Moreover, through paracrine mechanisms, c-kit^pos^ heterologous HLA nonmatched allogeneic CSC treatment preserved myocardial wall structure and attenuated remodelling by reducing myocyte hypertrophy, apoptosis and scar formation (fibrosis) [77]. Additionally, Marbán and colleagues [44] have tested the safety and efficacy of using allogeneic, mismatched cardiosphere-derived cells (CDCs) in infarcted rats. Rats underwent permanent ligation of the LAD coronary artery and two million CDCs or vehicles were intramyocardially injected at four sites in the peri-infarct zone. Three weeks post-MI, animals that received allogeneic CDCs exhibited smaller scar size, increased infarcted wall thickness and attenuation of LV remodelling. Allogeneic CDC transplantation resulted in a robust improvement of fractional area change (~12%), ejection fraction (~20%) and fractional shortening (~10%), and this was sustained for at least six months. Furthermore, allogeneic CDCs stimulated endogenous regenerative mechanisms (cardiomyocyte cycling, recruitment of c-kit^pos^ eCSCs, angiogenesis) and increased myocardial VEGF, IGF-1 and HGF [44]. The positive data of these tests also in large animals were the basis to the first-in-man clinical trial testing the feasibility of allogeneic cardiac progenitors for ST-elevation MI [78,79]. Accordingly, allogeneic cardiosphere-derived cells and mesenchymal stem cells were also tested in clinical trials [80].

Communication between adult stem cells can occur through direct contact or the release of soluble factors, as chemokines, cytokines, growth factors and hormones [50,51,52,81,82]. These factors may act in an autocrine and an endocrine way reaching target cells through biological fluids [81,82]. In addition to these conventional communication ways, based on single molecules, cellular communication is also mediated by the release of complex structures, the so-called extracellular vesicles, which are an integral part of the intercellular tissue microenvironment [81,82]. Through this new communication system, cells acquire and ensure an additional way of regulating cell functions such as growth, proliferation, secretion of specific molecules, survival and interconnection in several pathophysiological conditions.

Among these well-established modalities of intercellular communication, emerging mechanisms and studies are focusing on exosomes. As above introduced, exosomes are indeed another piece of the puzzle describing the salutary effects of stem cell therapy in general as well as of CSCs in particular.

## 3. Biogenesis, Physicochemical Composition and Release Mechanism of Exosomes

As above introduced, cells can release complex structures, so-called extracellular vesicles (EVs) that, besides being an integral part of the intercellular microenvironment, represent another important mechanism to mediate cellular communication, also for regenerative phenomena [50,51,52,81,82]. While for hormones and neurotransmitters the secretion modalities and the mechanisms of action are generally well known, for the EVs, they are still incompletely understood.

**Figure 1:**
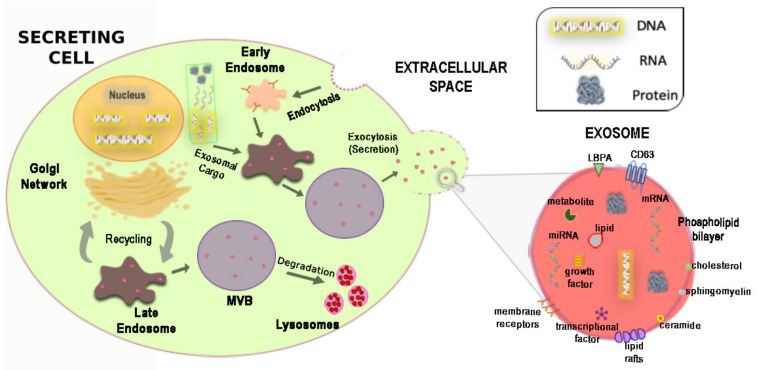
**Exosomes biogenesis and composition.***Left*: Representative cellular structure, showing the two main mechanisms of exosomes’ biogenesis: (1) the endocytosis process, in which vesicles from the plasma membrane that carry receptors from the cell surface, generate the early endosomes. During the endocytic process proteins, nucleic acids, lipids are internalized and incorporated into early endosomes, which then mature in the late endosomes and afterwards in multivesicular bodies (MVBs). These carrier vesicles are transported to, dock at and combine directly with the plasma membrane, releasing their contents in the extracellular environment, through exocytosis; (2) recycling of the endocytic pathway. Endosomes transport newly synthesized material from the Golgi complex and endocytosed material from the plasma membrane to various intracellular destinations. In particular, the early endosomes first and the MBV afterwards deliver cargoes to the lysosomal system for degradation. There, upon fusion of the limiting membrane of the MVB with lysosomes, causing remodeling of the phospholipidic bilayer, they become easily sensitive to degradation. *Right:* Representative image of the biochemical composition of exosomes. Exosomal vesicles contain a large repertoire of macromolecules, including nucleic acids, proteins, lipids, metabolites, transcriptional factors and growth factors. They present also a complex phospholipid bilayer membrane that has a protective role from external molecules and takes part in the interactions with the target cells.

EVs are transported by biological fluids and are capable of transferring genetic material, cytokines, adhesion molecules into neighboring cells or cells that are significantly distant from the place of production [50,51,52,81,82,83]. With the term, EVs have indicated all cell-derived vesicles released into the extracellular environment such as exosomes, membrane vesicles, apoptotic bodies, microparticles (released by platelets) and ectosomes (released by polymorphonuclear leukocytes) [84,85]. The EVs differ from each other for the origin, antigenic composition and also for the dimensions [84,85]. For a long time, it was thought that EVs were inert cellular debris, present in the blood or the interstitial spaces, and derived from damaged cells or the normal plasma membrane turnover [84] and only able to promote blood coagulation [84,85]. In 1967, Wolf called them “platelet-dust” [85]. It took 10 years since then for De Broe and colleagues to suggest a role of extracellular vesicles in cell physiology [86]. EVs were later isolated from most types of cells and biological fluids such as saliva, urine, amniotic fluid, breast milk, plasma and serum [82,87]. The main types of EVs, which have generated a significant interest but also some confusion in the literature, are exosomes and membrane vesicles; the latter originate by direct budding from the plasma membrane, with a calcium-dependent mechanism [87,88]. On the other hand, exosomes are small membrane-derived particles, with a dimension that varies from 30 to 150 nm [53] and characterized by an almost homogeneous shape [84]. Their biogenesis is the result of two main mechanisms: (1) the recycling of components derived from the endocytic pathway [89]; (2) the endocytosis process, in which vesicles are formed at the plasma membrane, generating the early endosomes [90] (Figure 1).

Exosomal formation, the sorting of the cargo proteins and their release require the “endosomal ordering complex necessary for transport” (ESCRT complex) [91] and other associated proteins such as Alix and Tsg101 [83,87,88,91]. This release mechanism can take place constitutively or in a regulated manner [92]. The biogenesis of exosomes begins with the formation of the endocytic vesicles from specialized regions of the plasma membrane [84,86,87] (Figure 1). These vesicles leave the membrane, merging with the early endosome. There, because of the acid environment, a part of the receptors is internalized, changing their conformation and releasing ligands, which are degraded by lysosomes [88,93,94]. Instead, the receptors and the ligands that remain attached, are conveyed to the late endosome, spherical vesicles, where the proteins are incorporated into intraluminal vesicles (ILVs) to generate the multivesicular bodies (MVBs) [94] (Figure 1). Subsequently, the MVBs are conveyed toward the plasma membrane and by merging with it, they release their contents in the extracellular environment. Once released, the vesicles are called exosomes [84,87] (Figure 1).

To date, the mechanisms underlying the formation of MVBs are yet incompletely defined, and it has been hypothesized that the ESCRT complex is the major, but not the only mechanism involved in the biogenesis of exosomes [91,94]. It appears that this process is also strongly influenced by the cell type and the conditions of the cells under examination [83]. After the ILVs become MVBs, the release into the extracellular environment requires a series of steps such as transport, docking and merging with the membrane [83,84,87,89]. Most of these mechanisms are not fully understood, although several recently published studies have tried to clarify them [91,93,95].

The formation of ILVs can be mediated by various factors such as the ESCRT protein complex, tetraspanins (CD9/CD63/CD81/CD82) [83,91] and some lipids such as LBPA and ceramide [83,91]. The intravesicular traffic that regulates the transport of MVBs to the plasma membrane can be mediated by numerous proteins belonging to the Rab family, such as RAB27, RAB11 and RAB35 [96,97], with calcium-dependent mechanisms. The fusion of MVBs with the plasma membrane appears to be mediated by SNAREs proteins [83]. Furthermore, it has shown a direct involvement of the transcription factor p53 in the exosomal secretion; p53 activation by UV radiation is followed by an increase in the effector protein Tsap6, with a consequent increase in the release of exosomes [98].

The release of membrane vesicles is a physiological phenomenon that occurs with a calcium-dependent mechanism, which triggers modifications of the actinic cytoskeleton at the level of the plasma membrane and the tubulin cytoskeleton [83,88]. The increase of calcium in the intracellular space, induced by an external signal, also determines the loss of the asymmetry of the phospholipid bilayer and the displacement of the phosphatidylserine from the inner side to the outer one of the membrane [81,82]. This causes the consequent separation of the vesicle from the membrane [81,82]. However, calcium is not the only second messenger involved in the mechanism of release of the exosomes, as indeed in different cell types it has been shown that the activation of PKC as well as of PKA can trigger the release of the EVs [99,100].

The exosome morphology is typically defined as “cup-shaped”. The biochemical composition of exosomes is made of a membrane of phospholipids that contains relatively high levels of cholesterol, sphingomyelin and ceramide [83,91,94] (Figure 1). Exosomal composition is a consequence of their formation through the endosomal compartment as well as the various mechanisms associated with their secretion. The composition of exosomes differs from MVs and source cells for lipid and protein contents, therefore different studies have identified proteomic and lipidomic differences [83,91,94].

The mechanism of exosome release may be also stimulated by cell stress, probably as a defensive mechanism [53,93,98]. This evidence brought exosomes to be the focus of several studies in these years. To date, it is known that practically all cells can release EVs [81,82]. Obviously, extracellular vesicles released by different cell types can have different antigenic compositions because of different phospholipids, proteins and other molecules that they express on the surface (Figure 1). After release, exosomes can influence the behavior of target cells in several ways. They can act as signaling modulators, transferring membrane receptors and other proteins to target cells, or modifying cell phenotype by horizontal transfer of genetic information [82,83,94]. Although the content of the exosomes reflects the cell from which they originated, there is selective targeting of the macromolecules included in them [94,95]. The mechanisms with which cytosolic constituents are recruited into exosomes remain to be defined but appear to involve the association of exosomal membrane proteins with chaperones, such as HSC70, which are found in exosomes from most cell types [95]. Specific proteins can be included or excluded from the EVs and therefore their expression pattern may differ from the cell surface from which they originated. MVBs are also sites of miRNA-loaded RNA-induced silencing complex accumulation and the fact that exosome-like vesicles are considerably enriched in GW182 and AGO2 implicates the functional roles of these proteins in RNA sorting to exosomes. To date, the function of the different miRNAs in exosomes represents the most considered subject of different studies. Thus, exosome-derived miRNAs could be considered novel and potential biomarkers of physiological and pathological conditions, regulating metabolism and providing a new mechanism of crosstalk between cells [101,102].

The peculiarity of exosomal miRNAs is the presence of the membrane that protects them from degradation by extracellular and circulating RNAse of the body fluids. Once released, exosomes can be degraded rapidly, thus freeing their cargo in the extracellular environment near the cells that released them, or they can circulate in and through different biological fluids and reach target sites far from their primary release site. These mechanisms explain why exosomes are found in all biological fluids, such as blood, urine, milk and cerebrospinal fluid.

On the other hand, exosomes are not able to interact with all cell types, but only with specific cells. The interactions with target cells are not fully elucidated, but it could occur through different mechanisms: (1) through binding to specific receptors expressed on the surface of target cells, which can trigger a signaling mechanism and the consequent formation of a multimolecular extracellular complex; (2) by direct fusion of its membrane with that of the target cell, thus immediately releasing its contents inside the cell; (3) through endocytosis. In the latter case, the endocytic exosome can remain segregated within endosomes and then merge with lysosomes to be degraded or can melt its membrane with the endosomal one to release its contents inside the cytoplasm of the target cell or by transcytosis can reach the extracellular space following the fusion of the endosome with the plasma membrane [103].

Among the target tissues of the exosomes, there is also the heart. Indeed, once released into the heart by different cell types, including exogenous transplanted stem and progenitor cells, exosomes exert cardiac protection. Mainly, for this reason, they have been proposed as elements that improve tissue environment after damage, a significant phenomenon to optimize survival and engraftment of cells after their transplantation [50].

Evidence that the exosomes released from different stem cell types, including resident CPCs can be used to improve microenvironment and to promote the regenerative potential of these cells themselves, has opened a new perspective for stem cell therapy for CVDs.

Finally, exosomes can be engineered to become a custom-made delivery tool to release specific bioactive molecules to specific cells within a specific tissue [95,104,105]. Nowadays, nanotechnology has indeed been showing increasing interest in exosomes, especially for their above-mentioned capability to target specific tissues to provide personalized cell-free therapies. Currently, two different approaches have been investigated: the first one exploits their intrinsic capability to bind specific recipient cell receptors (natural-targeting properties) because of their composition due to the tissues of origin, influencing their surface’s proteins, structure’s lipids and glycans [106]. On the other hand, the second approach consists of exosomes’ engineering through the functionalization of their surface architecture with ligands able to recognize specific tissues [107,108]. Personalized exosome–mimetic nanovesicles could thus represent a promising emerging application in the future as a novel drug delivery system. Indeed, nanotechnology is looking at exosomes as ideal targets for nanotherapy [109,110], pointing at modifying the exosomes isolated from tissue-specific cells that are supposed to be prone to act within the relative tissue of origin. These nano-based exosomes would incorporate specific genes or miRNA to be delivered to the recipient tissue (i.e., the myocardium) to modify the microenvironment and obtain repair and regeneration. It is indeed envisioned that exosomes can be used as nanoparticles for targeted delivery of several miRNAs that can target different cellular processes, from inflammation to immune responses and from cardio-protection to cardiac regeneration, representing a novel form of gene therapy [56,111].

## 4. CPC-Derived Exosomes and Their Application for Cardiac Regeneration

As above discussed, one of the limitations of cardiac cell transplantation in clinical studies has been that only a few of the transplanted cells undergo differentiation in the injured tissues, and certainly not enough to obtain direct tissue regeneration [112]. The regenerative potential of stem cell transplantation is indeed limited by their reduced migratory capacity (homing) and engraftment toward the target site and by the low resistance to a cytotoxic environment, as in the case of the heart tissue after myocardial infarction that causes massive cell death [112]. Furthermore, adult and pluripotent stem cells transplantation to obtain direct cardiac tissue remuscularization is severely hampered by the action of the immune system [113,114].

Since the interaction between adult stem cells with their niche and the surrounding microenvironment play a fundamental role in determining cellular behavior and fate [115], several studies are now focusing on CPC-derived exosomes as new cargoes for cardio-reparative and cardio-regenerative factors [50,52,53,116,117,118].

New evidence identifies exosomes as key components of the paracrine factors in both human [50,119] and mouse CPCs [116]. Exosome-derived from CPCs were first isolated for being positive for markers of the tetraspanin family (CD63, CD81 and CD9) [120], TSG-101 and Alix [91,93] (Figure 1). Mouse CPC exosomes have a high-level expression of GATA4-responsive-miR-451 and promote cardiomyocyte survival in vitro by inhibiting caspase 3/7 activation [116]. Accordingly, in vivo delivery of CPC exosomes in an acute mouse myocardial ischemia-reperfusion model inhibited cardiomyocyte apoptosis in comparison with PBS control [116]. Furthermore, oxidative stress increased miR-21 in mouse Sca1(+) CPCs-derived exosomes and the miR-21/PDCD4 axis has an important role in the antiapoptotic effect of CPCs-derived exosomes on cardiomyocytes in vitro [117]. In a rat model of ischemia-reperfusion injury, Agarwal et al. [118] demonstrated that CPC-derived exosomes can repair the infarcted heart, although their efficacy decreases sharply with donor age. They also showed that subjecting cells from older children to hypoxia restored the reparative potential of the exosomes and changed their miRNA profile. Cardiac functional improvements were associated with increased angiogenesis, reduced fibrosis and improved hypertrophy, resulting in improved cardiac function [118]. Accordingly, human CPCs derived from atrial appendage assemble exosomes highly enriched with miR-210, miR-132 and miR-146a-3p [119]. Exosome-derived miR-210 downregulated its known targets, ephrin A3 and PTP1b, inhibiting apoptosis in cardiomyocytic cells [119]. On the other hand, miR-132 downregulated its target, RasGAP-p120, enhancing tube formation in endothelial cells [119]. Infarcted hearts injected with human CPC-derived exosomes, but not from fibroblasts, exhibited less cardiomyocyte apoptosis, enhanced angiogenesis and improved LV ejection fraction compared with those injected with control medium [119].

**Figure 2:**
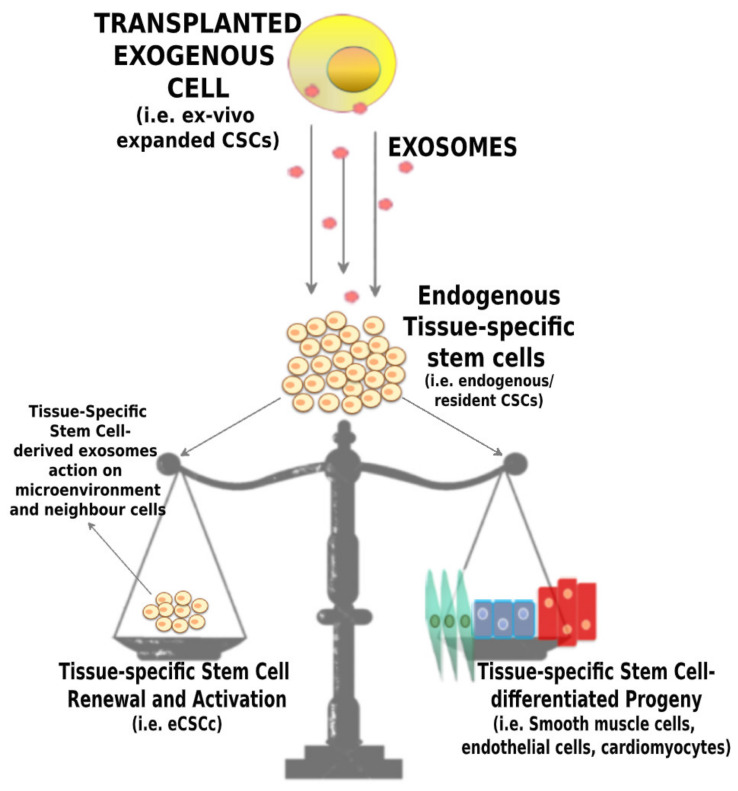
**Exogenously expanded Adult Stem Cell-derived exosomes act on endogenous tissue-specific adult stem cells (CSCs).** Schematic representation of the release of the exosomes derived from exogenous stem cell transplantation into the damaged organ (i.e., the heart), where they activate endogenous tissue-specific resident adult stem cells (i.e., CSCs). Exosomes may have a central role in maintaining the balance between self-renewal and differentiation, the two main characteristics of regenerative tissue-specific stem cells. Furthermore, upon stimulation from transplanted exogenous cells, the endogenous stem/progenitor cells (i.e., CSCs) can secrete exosomes that improve microenvironment, promote their regenerative potential, and regulate cell metabolism, providing a new mechanism of crosstalk between resident stem cells and neighbor cells.

**Figure 3:**
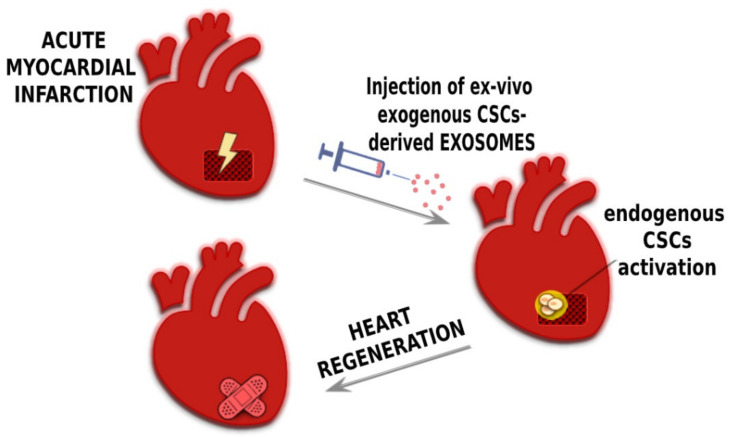
**Exosome-based Cell-free approach for cardiac regeneration.** Exosomes represent novel potential protective and regenerative cell-free therapies, carrying and delivering mRNAs, miRNAs and proteins to the damaged heart tissue, interacting with endothelial cells, cardiomyocytes, smooth muscle cells, fibroblasts and resident cardiac/progenitor cells within the heart upon transplantation, resulting in enhanced resident cardiac stem cell activation/expansion, cardiomyocyte survival and new formation, angiogenesis and modulation of cardiac inflammatory response, thereby increasing endogenous tissue repair/regenerative response to injury.

It appears that exosomes derived from CPCs may have a central role in maintaining the balance between self-renewal and differentiation of CPCs themselves, the two main characteristics that characterize stem cells [54,114,115,121] (Figure 2). Thus, exosomes derived from CPCs may improve the activation of the regenerative potential of the endogenous pool of cardiac tissue-specific stem/progenitor cells by transferring signal molecules directly within their niche and the myocardial microenvironment [121,122]. Moreover, exosomes derived from CPCs may release in the extracellular environment genetic information, modulating endogenous stem cell plasticity and tissue regeneration after damage [119,123,124] (Figure 3). Exosomes may also stimulate the angiogenesis process, are cytoprotective and modulate inflammatory and apoptotic processes [125]. Overall, this type of cell-to-cell communication has the aggregate effect of ameliorating the tissue microenvironment, stimulating resident CPC proliferation and differentiation [5,6] (Figure 3). Importantly, a better environment may improve the survival of transplanted CPCs activating pro-survival kinases and inducing a glycolytic switch in recipient host CPCs [126]. In a rat model of ischemia-reperfusion injury, Ciullo and colleagues [126] showed that the systemic administration of exosomes (genetically engineered to overexpress CXCR4–Exo^CXCR4^) derived from CXCR4-overexpressing (a transmembrane receptor—CD184) CPCs improve heart function. The expression of chemokines in infarcted myocardium is upregulated due to the activation of hypoxia-induced factor-1 (HIF-1). SDF-1α (a member of the CXC chemokine family overexpressed in infarcted heart post-MI) binds the CXCR4 receptor, acting as a potent chemoattractant for CXCR4 expressing cells including circulating progenitor cells. The Exo^CXCR4^ are more bioactive in the infarcted zone than naturally occurring exosomes (Exo^CTRL^) injected via tail-vein, confirming their superior homing and cardioprotective properties in the damaged heart.

Thus, thanks to the repertoire of macromolecules that they contain, exosomes derived from the CPCs may improve cardiac function and may reduce fibrosis after myocardial damage [119,121,122,123,124,127].

Another important evidence is correlated to the pro-regenerative effect of miRNAs released from CPC-derived exosomes, in response to differential myocardial stress and damage [39,40,41,117,118,119,121,122,123,124,127,128]. To date, this evidence that exosomes contain miRNAs has acquired a growing interest in the scientific community because it seems that these small RNA molecules may have a significant impact on cell and tissue homeostasis as well as in cellular phenotype specification [128,129]. Several exosomes-derived miRNAs were found to be upregulated in patients with different heart diseases when compared to those detected in exosomes derived from healthy control subjects [129,130]. Furthermore, the presence of exosomal miRNAs in the plasma and the alterations of their levels following myocardial infarction is a reproducible finding, paving the way to their use as biomarkers for myocardial infarction diagnosis and risk stratification [131]. In a novel rat model of Doxorubicin/Trastuzumab (Dox/Trz) cardiotoxicity, which mimics protocols used in clinical practice, it was demonstrated that systemically delivered CPC exosomal miRNAs exert protective effects, decreasing ROS levels normally higher in oncologic patients treated with anthracyclines and Trz. CPCs’ exosomes used were markedly enriched in miR-146a-5p, the most highly upregulated miR in human CPC exosomes. Furthermore, miR-146a-5p target genes encode signaling mediators of inflammatory and cell death axes. The in vivo systemic administration of these modified exosomes prevents myocardial fibrosis, LV dysfunction and increases iNOS levels, counteracting Dox/Trz negative effects [132]. Xiao and colleagues showed that CPCs secrete a higher quantity of exosomes in stressful conditions. Analyzing CPC-derived EVs, they identified among others an exosomal miRNA (miR-21) that was upregulated. In particular, miR-21 acts on PDC4 (programmed cell death 4) with an antiapoptotic effect in vitro. This study demonstrates that exosomal miR-21 derived from CPCs prevent cardiomyocytes apoptosis, protecting myocardial cells against oxidative stress through the inhibition of its target PDCD4 [117]. Finally, moving from small to large animals, CDC exosomes decrease scarring, halt adverse remodeling and improve LVEF in porcine acute and chronic myocardial infarct [133].

Overall, exosomes are novel potential cell elements regulating cell metabolism at tissue levels but also at distance, providing a new mechanism of crosstalk between cells in specific microenvironments and in particular between resident stem/progenitor cells [95]. The exosome content has a significant potential to be developed as new biotechnological and pharmacologic agents for regenerative medicine but also as diagnostic/prognostic tools for laboratory medicine.

## 5. Shortcomings of the Clinical Use of Exosomes for Cardiac Regenerative Therapies

Although exosomes can mediate the paracrine effect of stem cells and their uses seem to be beneficial, their administration has shown some restrictions in terms of pharmacokinetic parameters. Even if these nanovesicles result in a low immune response, some routes of administration such as the parental one, lead to a rapid clearance because of the earlier macrophages’ uptake. For this reason, several strategies have been investigated to overcome this limitation, and hydrogels have been thought of as suitable 3-D scaffolds for exosome delivery [107,134]. It was demonstrated that the use of a modified and bioactive hydrogel increases the stability of miRNAs and proteins in exosomes, facilitating their therapeutic effects in vivo with a more simple and effective approach [135].

Accordingly, Liu and co-workers have exploited *in situ* administration of exosomes into infarcted rat hearts; these exosomes were isolated from cardiomyocytes-derived iPS, loaded into a collagen-based hydrogel with stem-cell-specific miRNAs, known to modulate different cardiomyocyte molecular networks. This approach allowed a higher bioavailability due to a sustained and prolonged exosome release, with a consequent infarct size reduction, improved cardiac functions and a decreased CM apoptosis [42]. Moreover, in a rat model of acute MI, beneficial effects on hemodynamic heart function and vascular proliferation in peri-infarct zone four weeks after MI have been reported by Chen and co-workers as a result of the administration of exosomes (uniform in size and protein content) loaded into a modified hyaluronic acid hydrogel in vitro, showing a time-release over 21 days [136]. Finally, the route of exosomes administration may represent a further limitation for the clinical use of CPC-derived exosomes in CVD. Indeed, the intracoronary administration of CDC-derived exosomes in pigs with acute and chronic MI was ineffective, while their intramuscular local release exerted beneficial cellular, anatomical and functional effects [133].

## 6. Conclusions and Future Perspective

Adult CSCs constitute an essential cell element within the myocardial tissue microenvironment and are an important regenerative/reparative cell reservoir to be exploited for the replacement of cells lost as a result of damage or aging [11,13,19,21,54,56,57,58]. Since its inception, the goal of regenerative cardiology has always been the search of a population of cells that could be safely transplanted to the patients determining an effective remuscularization of the damaged organ [11,13,19,21,54,56,57,58]. Yet the presence of a tissue-specific endogenous regenerative process mastered by CSCs opens up the future to regenerative therapies that do not depend on direct cardiac regeneration from the transplanted cells but rather on the paracrine effects of the transplanted cells on the endogenous regenerating agents.

Stem-cell-based regenerative biological processes such as cell growth, proliferation and differentiation are finely tuned by cell-to-cell communication events between tissue-specific stem/progenitor cells within their niche, the environment surrounding them and circulating factors in the environment itself [122]. Harnessing the cellular and molecular basis of this cell-to-cell communications will be pivotal for novel cardio-regenerative therapeutics. Exosomes are primary players of these cell-to-cell communications transferring “paracrine factors” acting as signaling molecules within tissue microenvironment [137]. On this basis, exosomes offer unique opportunities for the development of new therapies, representing promising cell-free biotechnological therapeutics for myocardial repair and regeneration. Exosomes are more stable than cells under various physiological conditions and are to a certain degree immune-privileged. Moreover, exosomes can be stored long-term, making them ideally suited for therapeutic interventions. However, an important issue that remains to be elucidated is how exosomes pack their cargo and whether each and every exosome from the parent cell type cultured under the same conditions has similar content. In other words, it will be important to establish whether the bulk cell culture of CSCs may end up with a heterogeneous exosome formation. The latter might be avoided by the use of clonal CSCs. CSCs derived from heart failure patients as well as aging CSCs are known to be functionally impaired, and exosomes derived from such cells may already have compromised ability. Therefore, careful examination of CSC-derived exosome biology is required. Exosomes have a very short half-life and are quickly taken up by the target cells, limiting their effect to persist for a certain amount of time. Repeated injections may be something that needs to be developed in the future to enhance the effects of exosome therapy [138]. Similarly, exosome target selection is another potential area of consideration for the successful implementation of exosome therapy. The myocardium consists of cardiomyocytes, endothelial cells, stem cells, fibroblasts, and other interstitial cells. Exosomes may instigate opposite or similar responses in different target cells, influencing the outcome of the therapy, and require careful assessment and validation [138]. The latter might be mitigated by nanoencapsulation of exosomes to become super selective in their specific cell target.

Overall, the results of recent experimental studies suggest that CSC-derived exosomes possess great therapeutic potential that might overcome the shortcomings of cell and stem cell therapy and could open new frontiers in regenerative cardiovascular medicine. However, their role in cardiac regeneration needs further investigation to validate them as both biomarkers and as a therapeutic option.
